# COVID-19: the urgency of the doable for an African scientific leadership

**DOI:** 10.11604/pamj.2020.37.44.24401

**Published:** 2020-09-10

**Authors:** Francois-Xavier Mbopi-Keou, Jean-Emmanuel Pondi, Laurent Bélec

**Affiliations:** 1 The University of Yaounde I, Yaounde, Cameroon,; 2 The Institute for the Development of Africa (The-IDA), Yaounde, Cameroon,; 3 UNAIDS Scientific and Technical Advisory Committee (STAC) and The Board of Health Innovation Exchange, Geneva, Switzerland,; 4 The International Relations Institute of Cameroon (IRIC), Yaounde, Cameroon,; 5 Hôpital Européen Georges Pompidou, Université de Paris, Paris Sorbonne Cité, Paris, France

**Keywords:** COVID-19, the urgency of the doable, African scientific leadership

## Abstract

While Africa has long seemed paradoxically partly spared from the COVID-19 epidemic that is engulfing the rest of the planet, we are witnessing an upward surge in the dynamics of the epidemic across the continent. How to account for this unprecedented reversal of the COVID-19 epidemic situation in Africa, initially delayed compared to other continents, then apparently contained and now difficult to control? In our opinion, at least two factors play a major role in the current spread of the COVID-19 epidemic in Africa: the structural inadequacy of health systems and the serious deficiencies in the African approach to the response. We believe that political decision-makers must assume their responsibilities and inform their decisions on the basis of scientific facts, taking into account the specificities of our approaches to life, the only ones capable of containing fears and participating in the optimal management of crises and ultimately in the development of African nations. If Africa fails, the impact could be catastrophic not only from a health standpoint, but also from an economic standpoint and therefore from an overall human perspective. The great nations, like the great civilizations, know how to transform apparent challenges into undeniable opportunities to rise up. African scientists will similarly rise up, to meet the challenges of the COVID-19 epidemic.

## Opinion

While Africa has long seemed paradoxically partly spared from the COVID-19 epidemic that is engulfing the rest of the planet, we are witnessing an upward surge in the dynamics of the epidemic across the continent. According to the African Center for Disease Prevention and Control (or Africa Centers for Disease Control, Africa CDC, headquartered in Addis Ababa, Ethiopia), the continent recorded on Thursday June 18^th^, 2020 268,391 proven cases of COVID-19 and 7,217 overall deaths due to the disease. South Africa, Egypt and Nigeria are the countries most affected by the pandemic [1]. The upsurge in community contaminations now makes some experts fear the worst. How to account for this unprecedented reversal of the COVID-19 epidemic situation in Africa, initially delayed compared to other continents, then apparently contained and now difficult to control? In our opinion, at least two factors play a major role in the current spread of the COVID-19 epidemic in Africa.

**The structural inadequacy of health systems:** the fact that epidemics are more likely to occur in poor places with weak health systems does not help. The absence of hygiene or sanitation regulations or education, as well as the high density of urban African populations, are all factors that may increase the risk of epidemic spread. We note that some of the African countries which had set up containment measures are starting to lift them. If one acknowledges that these countries have carried out a relevant risk analysis, that does not provide them with the mastery of the dynamics of the epidemic, nor with the capacity to handle additional cases. There is already a lesson to be learned from the current crisis. The weakness of the health systems in Africa, with corollary an enormous amount of unmet needs for the user of the health sector. This has *de facto* negatively impacted on the preparation of African countries to face the new COVID-19. In addition, the strategic need to detect index cases of COVID-19 and to trace contact cases is weakened by the limited capacities for diagnostic tests in Africa [2].

**The serious deficiencies in the African approach to the response:** managing an unprecedented epidemic like that of SARS-CoV-2 infection requires multidisciplinary expertise, particularly in the medical as well as in the social sciences [3]. However, this expertise seems to have been largely lacking since the beginning of the epidemic in Africa. It is obvious that the under-involvement of African expertise in the COVID-19 crisis is more broadly part of the already well-recognized flight of African brains to other continents where their skills are better recognized and utilized. In other words, African expertise, although found in the diaspora, may have been largely lacking in Africa. Modern means of distance communication could have favored the recruitment of expatriate African experts in crisis management units, even if this approach has not been sufficiently explored. More generally, African expertise has been too infrequently included in operational decision-making teams [3]. Sometimes the genuine experts have not sufficiently been consulted. In any case, the combination of the relative scarcity of African expertise and its lack of sufficient mobilization have impoverished the management of the health crisis. Fortunately, international and even inter-African cooperation in health and the epidemic has enabled a more rational approach to managing the COVID-19 epidemic. Thus, the sub-regional centers of competence around the African offices of the World Health Organization, or the African Center for Disease Prevention and Control with its headquarters within the African Union in Addis Ababa in Ethiopia, brought in elements of enhanced expertise, particularly in training health partners about the COVID-19 epidemic, producing diagnostic tests and even supplying essential drugs to deal with the crisis [2]. In some countries, the traditional African pharmacopoeia has been used fully by large proportions of the population. International expertise has certainly helped manage technical aspects of the COVID-19 epidemic. However, it has often been lacking to support political decisions around the management of the epidemic, remaining more in the more particularly technical fields than in the operational and strategic fields.

**The urgency of the doable for African scientific leadership:** now, proposals must emerge to optimize Africa's responses to the challenge of COVID-19. Upstream and to stem the exodus of African human resources of high scientific level which weakens the African continent, it is a question of setting up an incentive framework for their development in Africa by bringing them the conditions of possibility of effective work. It is necessary to provide the necessary infrastructure, in particular in terms of laboratory resources, supported by suitable operating resources and trained and sufficient human resources. It is important to adopt a multidisciplinary approach as the facets of an epidemic like COVID-19 are both so many and unique. Thus, the biomedical sciences and the social sciences will be in the forefront, without forgetting the know-how resulting from the accumulated knowledge in the field of traditional African pharmacopoeia. The need to model epidemics, to predict future trends and to deviate from the deleterious trajectories, will require high level epidemiologist mathematicians, using powerful computing means. In the background, since scientific work does not blossom into autarky, it will be necessary to deepen and multiply intra-national and inter-African exchanges in all areas of medical and scientific knowledge. We believe that political decision-makers must assume their responsibilities and inform their decisions on the basis of scientific facts, taking into account the specificities of our approaches to life, the only ones capable of containing fears and participating in the optimal management of crisis and ultimately in the development of African nations. If Africa fails, the impact could be catastrophic not only from a health standpoint, but also from an economic standpoint and therefore from an overall human perspective. The great nations, like the great civilizations, know how to transform apparent challenges into undeniable opportunities to rise up. African scientists will similarly rise up, to meet the challenges of the COVID-19 epidemic.
